# In Vitro Sensitivity of Neuroendocrine Neoplasms to an Armed Oncolytic Measles Vaccine Virus

**DOI:** 10.3390/cancers16030488

**Published:** 2024-01-23

**Authors:** Nikolai V. Scheicher, Susanne Berchtold, Julia Beil, Irina Smirnow, Andrea Schenk, Ulrich M. Lauer

**Affiliations:** 1 Department of Medical Oncology and Pneumology, Virotherapy Center Tübingen (VCT), Medical University Hospital, 72076 Tübingen, Germany; nikolai.scheicher@med.uni-tuebingen.de (N.V.S.);; 2 Department of General Pediatrics, Hematology and Oncology, University Children’s Hospital, Eberhard Karls University Tübingen, 72076 Tübingen, Germany; 3 German Cancer Consortium (DKTK), Partner Site Tübingen, a Partnership between DKFZ and University Hospital Tübingen, 72076 Tübingen, Germany

**Keywords:** virotherapy, measles vaccine virus, combination therapy, super cytosine deaminase, neuroendocrine neoplasm, everolimus

## Abstract

**Simple Summary:**

Although a variety of treatment options are available for neuroendocrine neoplasms (NENs), amongst others, including systemic chemotherapy, radiation with the radiosensitizing CAP/TEM regime, the mTOR inhibitor everolimus, the multi-kinase inhibitor sunitinib, or immune checkpoint inhibitors, treatment efficacy is limited and the prognosis for this disease is still very poor. In this situation, oncolytic virotherapy with efficacy-enhanced second-generation measles vaccine virus MeV-SCD could be a promising therapeutic option and may open up a new platform for NEN patients by providing further combination therapies with existing therapeutics such as everolimus or immune checkpoint inhibitors.

**Abstract:**

Neuroendocrine neoplasms represent a heterogenous group of rare tumors whose current therapeutic options show only limited efficacy. Oncolytic viruses exert their mode of action through (onco-)lysis of infected tumor cells and the induction of a systemic antitumoral immune response in a virus-induced inflammatory micromilieu. Here, we investigated the potential of our well-established second-generation suicide-gene armed oncolytic measles vaccine virus (MeV-SCD) in five human NEN cell lines. First, (i) expression of the MeV receptor CD46 and (ii) its correlation with primary infection rates were analyzed. Next, (iii) promising combination partners for MeV-SCD were tested by employing either the prodrug 5-fluorocytosine, which is converted into the chemotherapeutic compound 5-fluorouracil, or the mTOR-inhibitor everolimus. As a result, MeV-SCD was found to kill all NEN tumor cell lines efficiently in a dose-dependent manner. This oncolytic effect was further enhanced by exploiting the prodrug-converting system, which was found to be highly instrumental in overcoming the partial resistance found in a single NEN cell line. Furthermore, viral replication was unaffected by everolimus, which is a basic requirement for combined use in NEN patients. These data suggest that MeV-SCD has profound potential for patients with NEN, thus paving the way for early clinical trials.

## 1. Introduction

Neuroendocrine neoplasms (NENs) refer to a group of relatively rare and heterogeneous tumors deriving from hormone-producing (endocrine) cells that can occur at different anatomical sites of the body. NENs have been described in the central nervous system, the respiratory tract, the gastrointestinal (GI) tract, the skin, the breast, and the urogenital system. However, the most common primary tumor sites are the GI (62–67%) and the respiratory tract (22%) [1]. Despite being a relatively rare tumor entity accounting for around 0.5% of all newly diagnosed malignancies, steadily and rapidly rising incidence and prevalence have been observed lately [2,3,4,5].

Based on clinical behavior, histology, and proliferation rate, NENs can be subdivided into two groups: Well-differentiated neuroendocrine tumors (NETs) and poorly differentiated neuroendocrine carcinomas (NECs) [6,7]. For the most frequent gastroenteropancreatic (GEP)-NETs, well-differentiated cases can be further categorized according to the 2019 WHO classification of tumors of the digestive system depending on their respective Ki-67 index as G1 (low grade, Ki-67 < 3%), G2 (intermediate grade, Ki-67 3–20%), and G3 (high grade, Ki-67 > 20%), whereas NECs per definition have a Ki-67 index > 20% [6].

Approximately one-fifth of the NEN patients already have metastatic disease at first presentation. However, the risk of metastasis varies considerably depending on the primary site, with GEP-NETs being most common in advanced disease stages and the liver being the most prevalent site of metastasis [8]. The 5-year-survival rate of patients with metastatic disease is only in the range of 19–38%, constituting a relatively poor prognosis [8]. In this situation, where curative surgery is lacking, current therapeutic options are somatostatin analogs, systemic chemotherapy, peptide receptor radiotherapy, radiation, mTOR-inhibitor everolimus, multi-kinase inhibitor sunitinib, interferon, or debulking surgery, all of which are associated with limited efficacy and poor prognosis [9,10].

Oncolytic viruses (OVs) are emerging as a new class of anticancer agents, being successfully used in the treatment of various malignancies [11,12]. The prototype is the viro-immunotherapeutic agent T-VEC (talimogene laherparepvec)/IMLYGIC^®^, which has been clinically approved in 2015 by both the FDA and EMA for the treatment of advanced melanomas and has shown encouraging results in ongoing clinical surveillance [13,14,15,16]. Regarding their mechanism of action, OVs selectively (onco-)lyse tumor cells and subsequently trigger a virus-antigen-enhanced anti-tumoral immune reaction in a virus-induced highly inflammatory tumor microenvironment, resulting in a strong and long-lasting systemic anti-tumoral immune response [17,18]. This is caused by dendritic cell activation as well as antigen presentation, thus priming T cells [19].

Although a large variety of oncolytic viruses have been tested preclinically for their potential oncolytic effect on NENs [20,21], only two of them have been investigated in clinical trials. One of them is the Seneca Valley virus (SVV001), which was evaluated in both children and adults for various cancers with neuroendocrine features. However, despite its high selectivity for NEN tumor cell lines [22], clinical trials have failed to show objective responses [23,24,25]. Another ongoing clinical phase I/II study (NCT02749331) investigates the effect of a genetically engineered triple-modified adenovirus serotype 5 (AdVince) for the treatment of liver metastases caused by NENs. In this study, AdVince is administered either into the hepatic artery or ultrasound-guided into the liver metastasis and is designed to specifically target NEN cells by putting the adenoviral E1A gene, which is essential for adenovirus replication, under the control of the human chromogranin A promoter [26]. Moreover, the insertion of the liver-specific miRNA 122 into the adenoviral genome downregulates remnant viral replication in healthy hepatocytes, thereby reducing the side effects and potential liver toxicity of the treatment [27].

In our laboratory, we now have employed two additional viral vectors for treatment of a panel of five human NEN cell lines: (1) GLV-1h68, a marker-gene encoding recombinant vaccinia virus, currently clinically developed as olvimulogene nanivacirepvec (Olvi-Vec) in ovarian cancer patients (NCT05281471), and (2) T-VEC, the first-generation recombinant herpes simplex virus type-1, being licensed so far only for advanced melanomas.

While T-VEC has already been demonstrated to successfully reduce the mass of five human NEN cell lines in an MOI-dependent manner at strikingly low MOIs [28], there was a single NEC cell line in which a partial resistance to T-VEC was observed, which might be due to the slower viral growth kinetics observed in the respective NEC cell line.

Interestingly, the vaccinia virus GLV-1h68 has also been shown to reduce several NET and NEC cell lines in a dose-dependent manner: three of them proved to be highly permissive towards the chosen criteria, three were defined as intermediate permissive, and no resistant NEN cell line was found, which has been interpreted as a pretty good result [29].

Here, we have also tested a measles vaccine-based oncolytic virus (MeV), which constitutes a highly attenuated negative-stranded RNA virus from the paramyxovirus group. Since this virus has been used as a measles vaccine for more than 50 years, it has an excellent, proven safety profile. Furthermore, it has already been shown to exert a strong oncolytic effect on multiple solid tumor types [30]. Most notably, a case of complete remission after a single systemic treatment with MeV has been observed in a patient suffering from therapy-refractory multiple myeloma [31].

The oncolytic potential of first-generation marker-gene encoding MeV can be further enhanced by arming, e.g., with the suicide-enzymes cytosine deaminase and uracil phosphoribosyltransferase, also called super cytosine deaminase (MeV-SCD), converting the non-toxic prodrug 5-fluorocytosine (5-FC) into the chemotherapeutic compound 5-fluorouracil (5-FU). 5-FU is a classical chemotherapeutic drug that has been used for decades for various tumor entities and continues to be a therapeutic option for NEN if systemic chemotherapy is needed [10]. MeV-SCD has already been tested for various cancers and has shown profound oncolytic activity in both solid [32] and “liquid” [33] tumors. Its suicide gene function was proven to be able to overcome resistance towards unmodified measles vaccine virus [32]. It was shown that alterations in the innate immune defense play a decisive role in the susceptibility of the respective cell line [34].

This paper aims to show for the first time the antitumoral effect of the safe and well-characterized OV MeV-SCD against various NENs in a pre-clinical setting, evaluating if an oncolytic virotherapy could be a suitable treatment option for patients suffering from inoperable NENs. Furthermore, we determined if the OV-based tumoricidal effect could be enhanced by exploiting MeV-SCD’s inherent suicide gene function. In detail, infection rates of the respective NEN tumor types, quantification of MeV-SCD replication within the infected NEN tumor cells, and the oncolytic efficacy of MeV-SCD have been assessed. To the best of our knowledge, this is the first time that a measles vaccine virus construct has been evaluated for the treatment of NENs.

## 2. Materials and Methods

### 2.1. NET/NEC Cell Lines

In this study, a panel of one NEC and four NET cell lines from different anatomical origins was employed. The two pancreas-originating NET cell lines BON1 and QGP-1 were obtained from Dr. Renner (MPI Psychiatry, Munich, Germany) and the Japanese Collection of Research Bio-Resources Cell Bank (JCRB, Osaka, Japan), respectively. The H727 and UMC-11 lung NET cell lines were purchased from ATCC. HROC-57 cells are derived from a colon ascendens NEC and were obtained from PD Dr. Linnebacher (University Hospital Rostock, Germany). All five NET/NEC cell lines were characterized and described previously [35,36,37,38,39]. QGP-1, H727, and UMC-11 cells were maintained in RPMI-1640 medium (Gibco, Waltham, MA, USA) supplemented with 10% fetal calf serum (FCS, Biochrom, Cambridge, UK). BON1 cells were cultured in Dulbecco´s modified Eagle´s Medium (DMEM, Sigma-Aldrich, Burlington, MA, USA) supplemented with 10% FCS, and HROC-57 cells required DMEM/F12 medium (Gibco) with 10% FCS.

Vero cells (African green monkey kidney), which were used for titration assays, were obtained from the German Collection of Microorganisms and Cell Cultures (DSMZ, Braunschweig, Germany) and were cultured in DMEM supplemented with 10% FCS.

### 2.2. Measles Vaccine Virus

**Figure 1:**
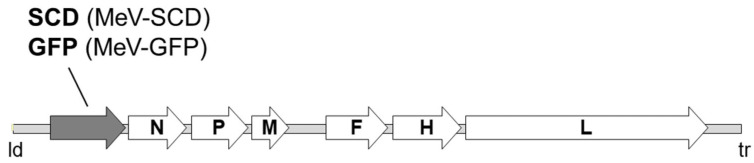
Schematic overview of the recombinant measles vaccine viral constructs MeV-SCD and MeV-GFP. Besides the viral genes *N* (encoding nucleoprotein), *P* (encoding phosphoprotein), *M* (encoding matrix protein), *F* (encoding fusion protein), *H* (encoding hemagglutinin protein) and *L* (encoding large protein), the recombinant MeV cDNA contains an additional transcription unit encoding SCD or GFP downstream of the leader (ld) position.

The two different measles vaccine viral constructs, MeV-SCD and MeV-GFP, were used; both originated from a commercially available original monovalent vaccine batch of measles virus (MeV) strain Schwarz (Mérieux^®^, Sanofi-Pasteur, Leimen, Germany). In both viral constructs, the transgene was inserted downstream of the leader (ld) position (Figure 1). In MeV-GFP, a gene encoding green fluorescent protein (GFP) was inserted to improve the tracking of viral infection [34]. The second viral construct was modified by inserting a gene encoding the prodrug-converting enzyme Super-CD (MeV-SCD). This suicide gene comprises a fusion protein of yeast cytosine deaminase and yeast uracil phosphoribosyltransferase that converts the prodrug 5-fluorocytosine (5-FC) into the chemotherapeutic 5-fluorouracil (5-FU) and facilitates further conversion into the cytotoxic compound 5-fluorouridine monophosphate (5-FUMP) [32,34,40,41,42].

### 2.3. Sulforhodamine B (SRB) Viability Assay

NET/NEC cells were seeded in 24-well plates and infected with MeV-SCD at the indicated multiplicities of infection (MOI). MOCK-treated cells (no virus, infection medium only) served as control. For infection, cells were washed once with PBS and then treated with a distinct MOI of MeV-SCD diluted in 250 µL Opti-MEM (Invitrogen, Darmstadt, Germany). At 3 h post-infection (hpi), the inoculum was removed and changed to standard medium with or without 1 mM of the prodrug 5-FC (Roche, Grenzach-Wyhlen, Germany). The remaining cell mass was measured at 96 hpi by using the SRB viability assay as described previously [28,43].

### 2.4. FACS Analysis

For detection of CD46 expression by flow cytometry, NET/NEC cells were washed with PBS, detached with accutase (PAA Laboratories, Cölbe, Germany), and diluted in FACS buffer (PBS containing 10% FCS). 5 × 10^5^ cells were treated with Gamunex to block Fc receptors before staining was performed as described previously [32]. Flow cytometry analysis was conducted on a FACSCalibur (Becton-Dickinson, Franklin Lakes, NJ, USA) using Cell Quest software (Becton-Dickinson). The mean fluorescence index (MFI) is defined as the arithmetic mean of CD46 divided by the arithmetic mean of isotype control.

For quantification of primary infection rates, NET/NEC cells were seeded in 6-well plates and infected with MeV-GFP (green fluorescent protein (GFP)-encoding MeV vector in which the GFP marker gene is placed at the same position as the SCD suicide gene in MeV-SCD) at different MOIs (0.1, 1, 10). At 3 hpi, infection medium was replaced by 2 mL of standard growth medium. At 24 hpi, the percentage of GFP-expressing cells was determined by flow cytometry as described before [32]. Therefore, cells were washed with PBS, detached with accutase (PAA Laboratories), and resuspended in 1 mL of FACS buffer and 3 mL of PBS. After centrifugation (5 min at 302× *g*) at RT, the pellet was resuspended in FACS buffer. Cells were fixed with 1.3% paraformaldehyde (Otto Fischar, Saarbruecken, Germany) and analyzed on a FACSCalibur (Becton-Dickinson) using the Cell Quest software (Becton-Dickinson).

### 2.5. Immunoblot Analysis

NET/NEC cells were seeded in 6-well plates and infected with MeV-SCD in 1 mL Opti-MEM at MOIs adjusted to the respective cell line-dependent sensitivity to virus infection. At 3 hpi, the inoculum was removed, cells were washed three times with PBS, and the medium was changed to standard growth medium. At 96 hpi, cells were washed with PBS and lysed in lysis buffer (50 mM Tris (pH 7.6), 150 mM NaCl, 1% Nonidet P-40). In order to release proteins, samples were additionally lysed through three freeze–thaw cycles. After centrifugation (10 min at 14,000 rpm, 4 °C), supernatants were transferred into new tubes, and protein concentrations were determined by a Bradford protein assay (Bio-Rad, Hercules, CA, USA). Proteins were separated by SDS-PAGE and transferred to a hydrophobic polyvinylidene difluoride (PVDF) membrane (Hybond-P, GE Healthcare, Waukesha, WI, USA) as described earlier [32]. Membranes were incubated with primary antibodies (anti-SCD; 1:1000; kind gift from Transgene S.A., Illkirch-Graffenstaden, France; and anti-Vinculin; 1:5000; Sigma Aldrich, St. Louis, MO, USA) and secondary antibodies (horseradish peroxidase-conjugated anti-rabbit, anti-rat, and anti-mouse) before proteins were detected with Amersham ECL Western blotting detection reagents (GE Healthcare, Buckinghamshire, UK).

### 2.6. Viral Growth Curve

NET/NEC cells were seeded in 6-well plates and infected with MeV-SCD in 1 mL Opti-MEM at MOIs adjusted to the respective cell line-dependent sensitivity to virus infection. At 3 hpi, the inoculum was removed, cells were washed three times with PBS, and medium was changed to standard medium with or without either 1 mM of the prodrug 5-FC or everolimus in concentrations adjusted to the respective cell line-dependent sensitivity (for all cell lines, 1 nM with the exception of UMC-11, where 0.25 nM was used). At 3, 24, 48, 72, and 96 hpi, cells were harvested by scraping into their medium. Quantification of viral particles (plaque-forming units (PFU)/mL) was performed after one freeze-thaw cycle via TCID_50_ titration on Vero cells according to the method of Kärber [44] and Spearman [45]. Since MeV-SCD lacks a fluorescent marker gene, infected cells were detected by immunofluorescence staining, as described earlier [32].

### 2.7. Statistical Analysis

Statistical analysis was performed with GraphPad Prism Version 9 (GraphPad Software Inc., San Diego, CA, USA). A Brown–Forsythe and Welch ANOVA and Dunnett’s multiple comparison test were used to determine the significance between the two treatment groups. Four different *p*-values were determined: *p* < 0.05 (*), *p* < 0.01 (**), *p* < 0.001 (***), and *p* < 0.0001 (****).

## 3. Results

### 3.1. Determination of CD46 Receptor Expression on the Surfaces of NET/NEC Cells

**Figure 2:**
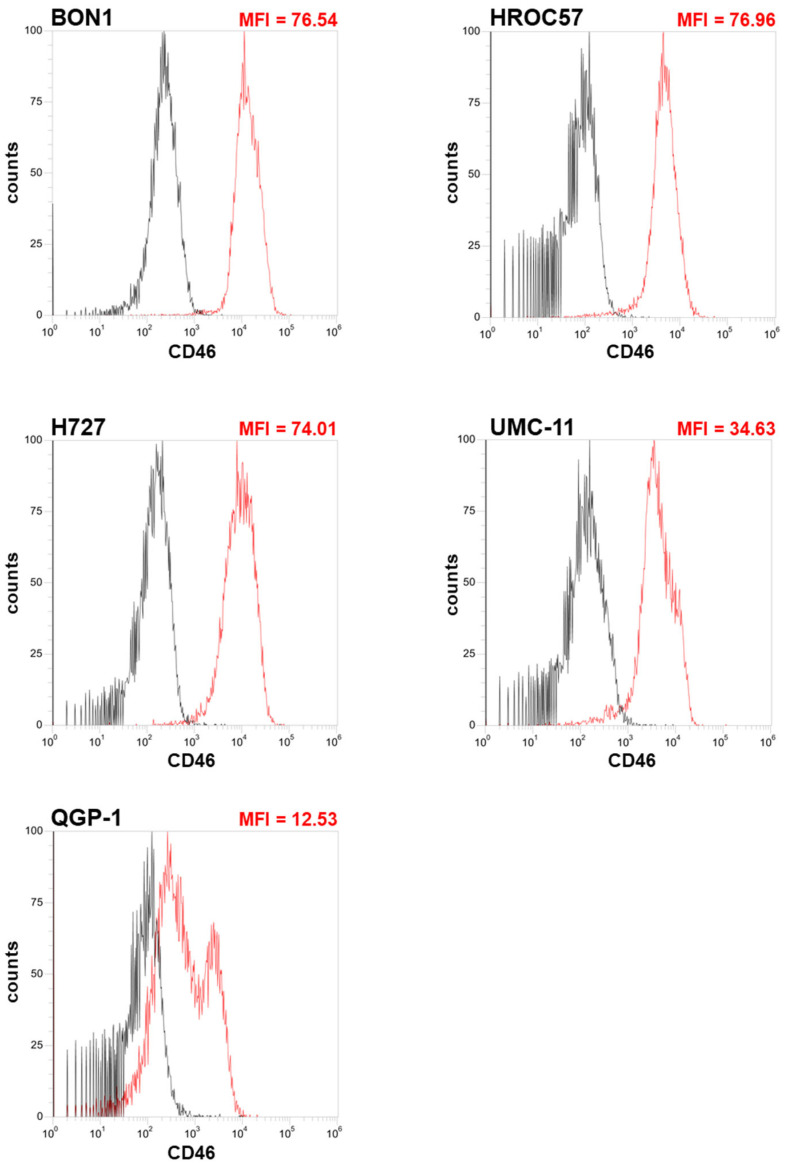
Determination of CD46 receptor expression on human NET/NEC tumor cell lines. The NET/NEC tumor cell lines BON1, HROC57, H727, UMC-11 and QGP-1 were stained with CD46 antibody (red histograms) or an isotype control (black histograms). Mean fluorescence was measured by flow cytometry. Mean fluorescence index (MFI) is the arithmetic mean of the CD46 receptor signal divided by the arithmetic mean of the isotype control signal. MFI 20 is considered as the minimum threshold required for syncytia formation.

In a first step, we analyzed CD46 receptor expression on NET/NEC cell surfaces using flow cytometry (Figure 2). CD46, which is ubiquitously expressed on all nucleated cells and is known to be overexpressed on various tumor entities, is the main route by which measles vaccine viruses can enter and therefore infect cells. There is a correlation between viral entry and the density of the CD46 receptor, while a certain threshold of a minimal mean fluorescence index (MFI) of 20 is required to ultimately lead to syncytia formation and cell death [46].

As a result, CD46 was found to be expressed on all NET/NEC cell lines tested. However, the CD46 density varied largely between the different cell lines, as depicted by the wide range of the mean fluorescence index (MFI) when comparing the respective NEN cell lines.

While NEN cell lines HROC57, BON1, and H727 were found to exhibit the highest MFIs (with values around 75), NEN cell line UMC-11 was tested with an intermediate MFI of 35, while QGP-1 displayed an MFI far below 20, being considered to be the minimum threshold for the achievement of syncytia formation, which is known to be a prerequisite for any productive replication of MeV vectors (Figure 2).

### 3.2. Assessing Primary Infection Efficiency of NET/NEC Cell Lines Using MeV-GFP

**Figure 3:**
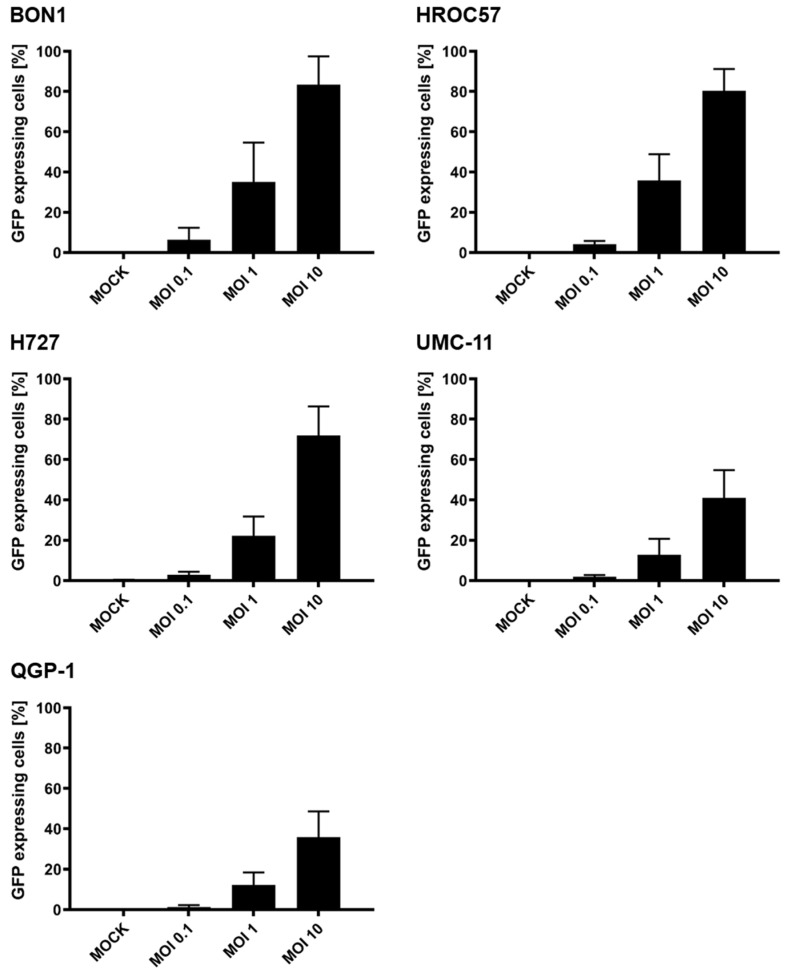
Primary infection rates of human NET/NEC tumor cell lines after infection with MeV-GFP. The NET/NEC tumor cell lines BON1, HROC57, H727, UMC-11 and QGP-1 were infected with the green fluorescent protein (GFP)-expressing measles vaccine virus MeV-GFP at different MOIs (0.1, 1, 10). At 24 hpi, the percentage of GFP-expressing cells was determined by flow cytometry. Mean and standard deviation of three independent experiments are shown. MOCK: untreated control. MOI: multiplicity of infection.

In the next step, we evaluated the primary infection rates of the different NET/NEC cell lines at 24 h post-infection (hpi) using a marker gene-encoding measles vaccine virus expressing green fluorescent protein (MeV-GFP), as displayed in Figure 3. MeV-GFP has its transgene in the same position as MeV-SCD, making it possible to transfer the results to the latter virus. MeV-GFP was used for this experiment since GFP facilitates the determination of the primary infection rates. Primary infection rates in this context refer to the percentage of infected cells in relation to all cells.

Interestingly, the three NEN cell lines with the highest expression of CD46 (BON1, H727, and HROC57; Figure 2) also showed the highest primary infection rates, with up to 80% of infected cells after 24 h at the highest MOI of 10 (Figure 3). Furthermore, the two cell lines with lower CD46 expression (UMC-11, QGP-1) were also less likely to be infected at 24 hpi, with only about 40% of infected cells at an MOI of 10.

### 3.3. Combating NET/NEC Cells with MeV-SCD

#### 3.3.1. Oncolytic Effect of MeV-SCD Monotherapy

**Figure 4:**
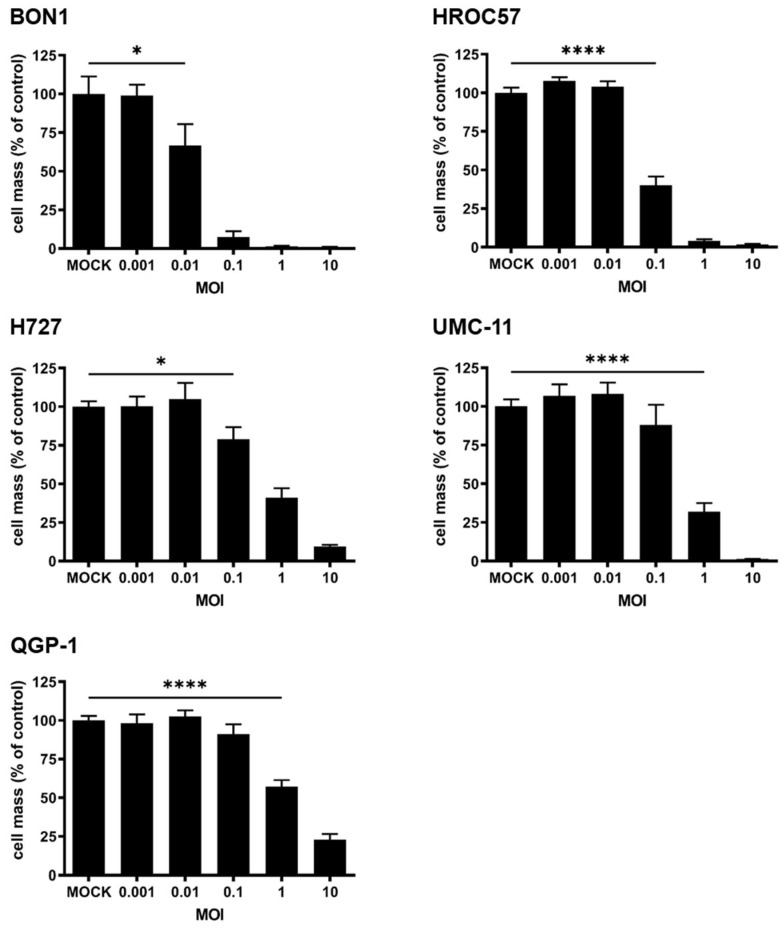
Viability of human NET/NEC tumor cell lines after infection with MeV-SCD. The NET/NEC tumor cell lines BON1, HROC57, H727, UMC-11, and QGP-1 were infected with the suicide gene-armed measles vaccine virus MeV-SCD at indicated MOIs. At 96 hpi, the remaining tumor cell masses were determined by SRB viability assay. Displayed are mean and standard deviation of one representative experiment performed in quadruplicates. MOCK: untreated control. MOI: multiplicity of infection. * *p* < 0.05, **** *p* < 0.0001.

To study the effect of MeV-SCD monotherapy on NET/NEC cell lines, we infected the respective cell lines with MOIs ranging from 0.001 to 10 (Figure 4). The remaining cell masses were determined at 96 hpi by the SRB viability assay. In analogy to other authors [32], we defined a cell line displaying a remaining tumor cell mass of more than 75% compared to MOCK infected controls when using an MOI of 1 as resistant, of more than 50% as partially resistant, while less than 50% of residual tumor cell mass applying the same MOI was classified as a permissive cell line to MeV-SCD infection.

As a result of our testing, four out of five NET/NEC cell lines proved to be permissive towards MeV-SCD-mediated oncolysis, while cell line QGP-1 showed a partial resistance according to our criteria (Figure 4). Nevertheless, when using a higher MOI of 10, strong tumoricidal effects could be observed in all tested NET/NEC cell lines, with remaining tumor cell masses of less than 25% when compared to MOCK-treated cells. However, the oncolytic effect of MeV-SCD monotherapy was strongest in both BON1 and HROC57 cells (Figure 4).

#### 3.3.2. Expression of SCD in MeV-SCD-Infected NET/NEC Cell Lines

**Figure 5:**
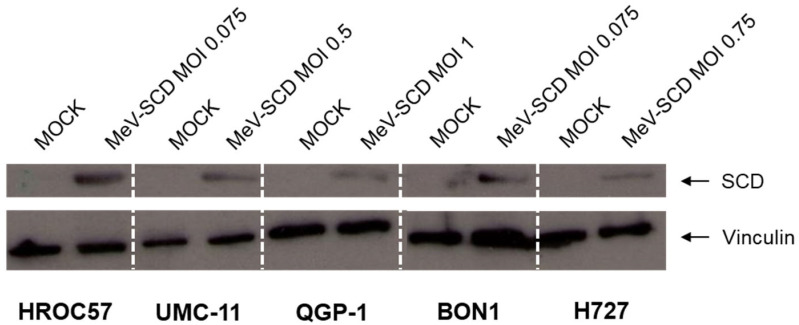
Immunoblot analysis of MeV-encoded SCD protein expression in human NET/NEC tumor cell lines. The NET/NEC tumor cell lines HROC57, UMC-11, QGP-1, BON1, and H727 were infected with the suicide gene-armed measles vaccine virus MeV-SCD at MOIs adjusted to the respective cell line-dependent sensitivity to virus infection. Whole-cellular protein extraction and immunoblot analysis with an anti-SCD antibody were performed at 96 hpi. Vinculin was used as a loading control.

As a prerequisite for the subsequent combination therapy with 5-FC, the first essential step was to check whether the SCD transgene is expressed in infected NET/NEC cell lines. Therefore, all five NEN cell lines were infected with MeV-SCD at MOIs adjusted to the respective cell line’s sensitivity towards viral infection, and immunoblot analysis was performed at 96 hpi (Figure 5; raw data can be found in Supplementary Figures S1–S3). In every tested cell line, the MeV-encoded SCD protein was detected only in infected cells, while there was no expression of the viral protein observed in MOCK-infected cells. The intensity of the protein band varied remarkably among the different tested cell lines. The highest intensity was observed in HROC57 and BON1 cells, which also had higher CD46 expression as well as higher primary infection rates compared to the other examined cell lines. Nevertheless, SCD was detected in every examined cell line, displaying a successful infection and functional transcription of the encoded gene, thus making it possible to exploit its suicide function in the following experiments.

#### 3.3.3. Cytotoxic Effect of 5-Fluorouracil (5-FU) Monotherapy

In the next experiment, we evaluated the susceptibility of the five NET/NEC cell lines in response to the well-known chemotherapeutic drug 5-FU, which is commonly used in the treatment of NENs if systemic chemotherapy is required.

**Figure 6:**
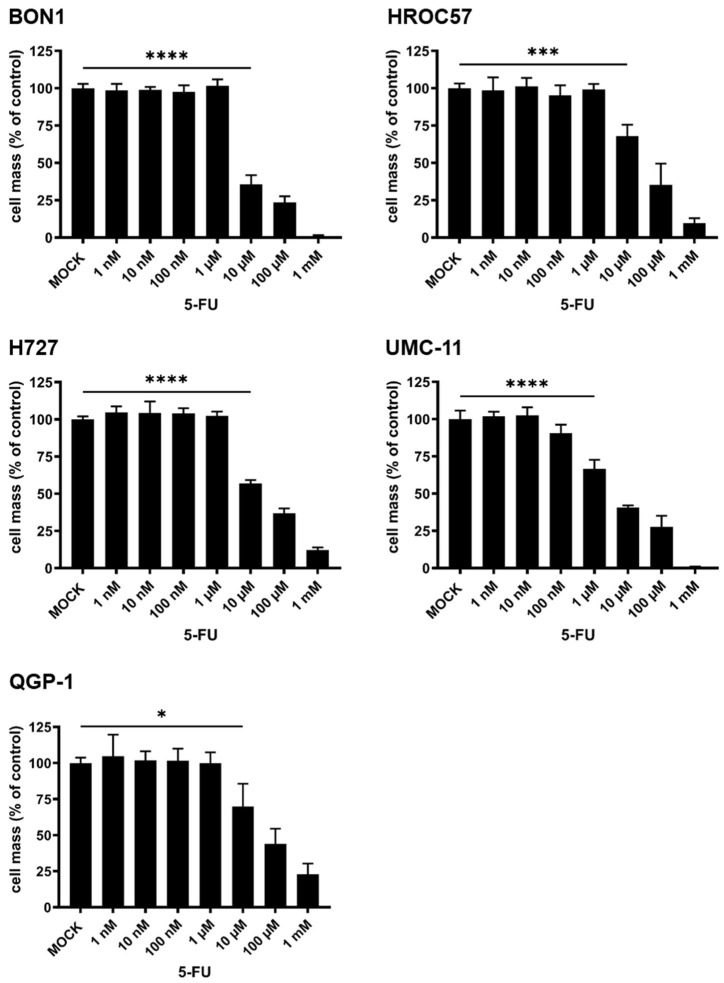
Susceptibility of human NET/NEC tumor cell lines to the chemotherapeutic compound 5-fluorouracil (5-FU). The NET/NEC tumor cell lines BON1, HROC57, H727, UMC-11, and QGP-1 were treated with rising concentrations of 5-FU, and the remaining tumor cell masses were measured at 96 h post-treatment (hpt) by SRB viability assay. Displayed are mean and standard deviation of two independent experiments performed in triplicates. MOCK: untreated control. * *p* < 0.05, *** *p* < 0.001, **** *p* < 0.0001.

For this purpose, the respective cell lines were treated with rising concentrations of 5-FU ranging from 1 nM to 1 mM, while the remaining cell masses were evaluated using the SRB viability assay 96 h post treatment (hpt) as displayed in Figure 6. All tested cell lines proved to be susceptible towards 5-FU treatment in a dose-dependent manner, with cell masses starting to decrease significantly at a concentration of 10 µM 5-FU, except in the cell line UMC-11, where as little as 1 µM 5-FU was already found to be sufficient to cause significant tumor cell death. While there were hardly any residual tumor cells when using 1 mM 5-FU in H727, BON1, UMC-11, and HROC57 cells, the effect was not quite as pronounced in QGP-1 cells (Figure 6).

#### 3.3.4. Exploitation of the SCD Prodrug-Converting System by Addition of 5-Fluorocytosine (5-FC) to MeV-SCD Therapy

Since all tested cell lines proved to be sensitive to both MeV-SCD and 5-FU monotherapy, we next evaluated the effect of MeV-SCD’s suicide function, which locally converts the prodrug 5-FC into the common chemotherapeutic 5-FU.

The five NET/NEC cell lines were infected with MeV-SCD at MOIs adjusted to their respective susceptibilities to the latter. At 3 hpi, the prodrug 5-FC was added, and the remaining cell masses were evaluated at 96 hpi.

**Figure 7:**
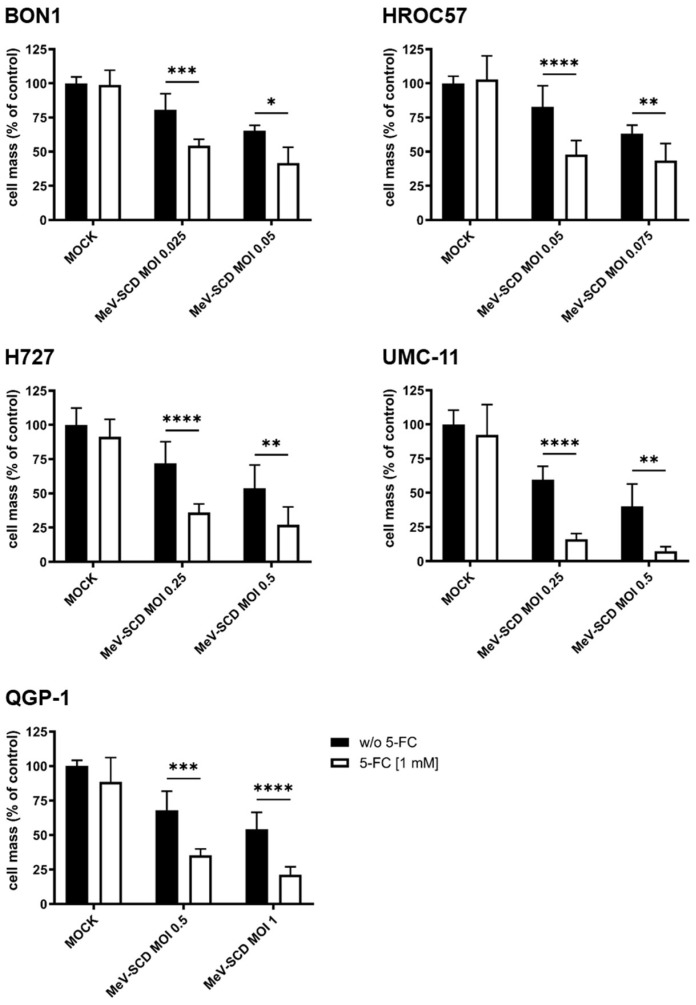
Remaining NET/NEC tumor cell masses after treatment with MeV-SCD and the prodrug 5-FC. The NET/NEC tumor cell lines BON1, HROC57, H727, UMC-11, and QGP-1 were infected with the suicide gene-armed measles vaccine virus MeV-SCD at MOIs adjusted to the respective cell line-dependent sensitivity to virus infection. At 3 hpi, 1 mM 5-FC was added, and cell viability was analyzed via SRB assay at 96 hpi. Displayed are mean and standard deviation of two independent experiments performed in quadruplicate. MOCK; untreated control. * *p* < 0.05, ** *p* < 0.01, *** *p* < 0.001, **** *p* < 0.0001.

As depicted in Figure 7, the exploitation of MeV-SCD’s suicide function led to further reduced tumor cell masses in all tested cell lines compared to MeV-SCD monotherapy. The strongest effect was seen in UMC-11 cells, where MeV-SCD monotherapy at MOI 0.25 reduced the remaining tumor cell mass to ~60%, while the addition of the prodrug led to less than 25% remaining viable tumor cells in comparison to MOCK-treated controls.

Strong effects were observed in the other cell lines as well. Importantly, cell line QGP-1, which showed a partial resistance according to our defined criteria with more than 50% remaining tumor cell mass in comparison to MOCK-treated controls when using an MOI of 1, now exhibited a residual tumor mass of only ~20% when exploiting MeV-SCD’s suicide function (Figure 7).

In conclusion, the exploitation of MeV-SCD’s inherent suicide function led to strongly enhanced oncolytic effects in all tested NET/NEC cell lines and was even able to overcome partial resistance phenomena in one cell line.

#### 3.3.5. Replication Characteristics of MeV-SCD When Combined with 5-FC

**Figure 8:**
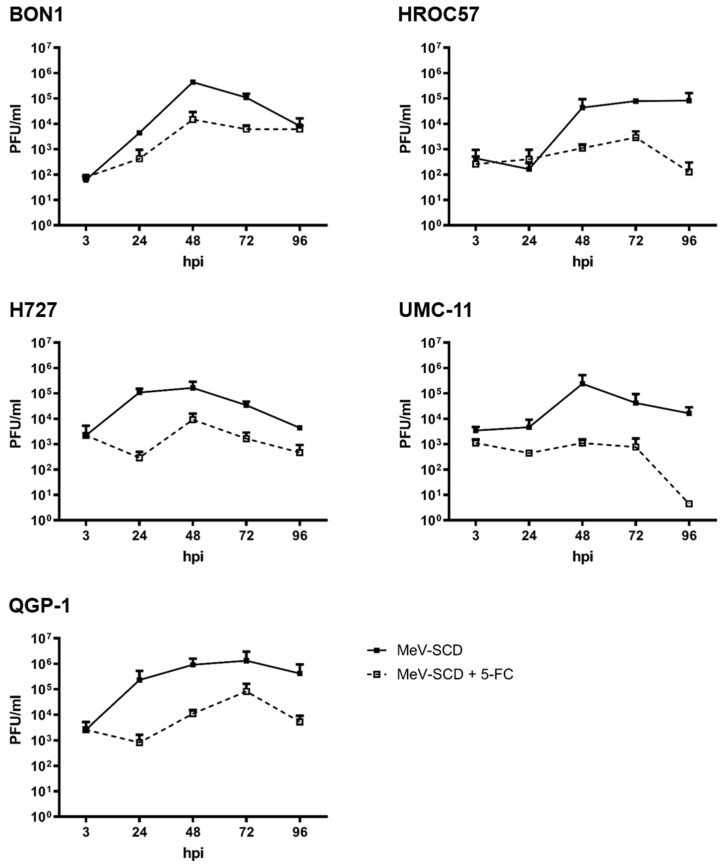
Replication of MeV-SCD in five human NET/NEC cell lines with and without addition of 5-FC. The NET/NEC tumor cell lines BON1, HROC57, H727, UMC-11, and QGP-1 were infected with the suicide gene-armed measles vaccine virus MeV-SCD at indicated MOIs, which were adjusted to the respective tumor cell line-dependent sensitivity. At 3 hpi, the prodrug 5-FC was added. Tumor cells and supernatants were harvested at 3, 24, 48, 72, and 96 hpi, and viral titers were determined by TCID_50_ titration. Displayed are mean and standard deviation of two independent experiments performed in duplicate.

While observing the replication characteristics of MeV-SCD in NEN cell lines after exploiting its suicide gene function by “simple” addition of 5-FC, it became evident that in every tested NEN cell line, the production of progeny virus particles was significantly decreased in comparison to single MeV-SCD replication (Figure 8). Changes in replication were observed as soon as 24 hpi and remained noticeable throughout the whole observation period of 96 h. A reduction in PFU of up to 3 log levels (UMC-11 96 hpi; Figure 8) was witnessed.

Bearing in mind that the addition of the prodrug led to significant decreases in viable tumor cell masses in all tested cell lines, this experiment indicates that the oncolytic effect of MeV-SCD not only depends on viral replication, but in addition, the conversion of 5-FC into the cytotoxic compound 5-FU plays a major role in tumor cell mass reduction.

### 3.4. Principles for Planned Clinical Combination Therapy of MeV-SCD with the mTOR-Inhibitor Everolimus

#### 3.4.1. Cytotoxic Effect of Everolimus Monotherapy

In a further experiment, the cytotoxic effect of everolimus monotherapy was studied on human NET/NEC cell lines. Everolimus, which serves as a mTOR-inhibitor and is used in clinical routine for a variety of different indications, was evaluated for the treatment of neuroendocrine neoplasia in the so-called RADIANT trials [47]. There it has shown beneficial results in patients suffering from NEN, making it one of the pillars in the treatment of NENs today.

**Figure 9:**
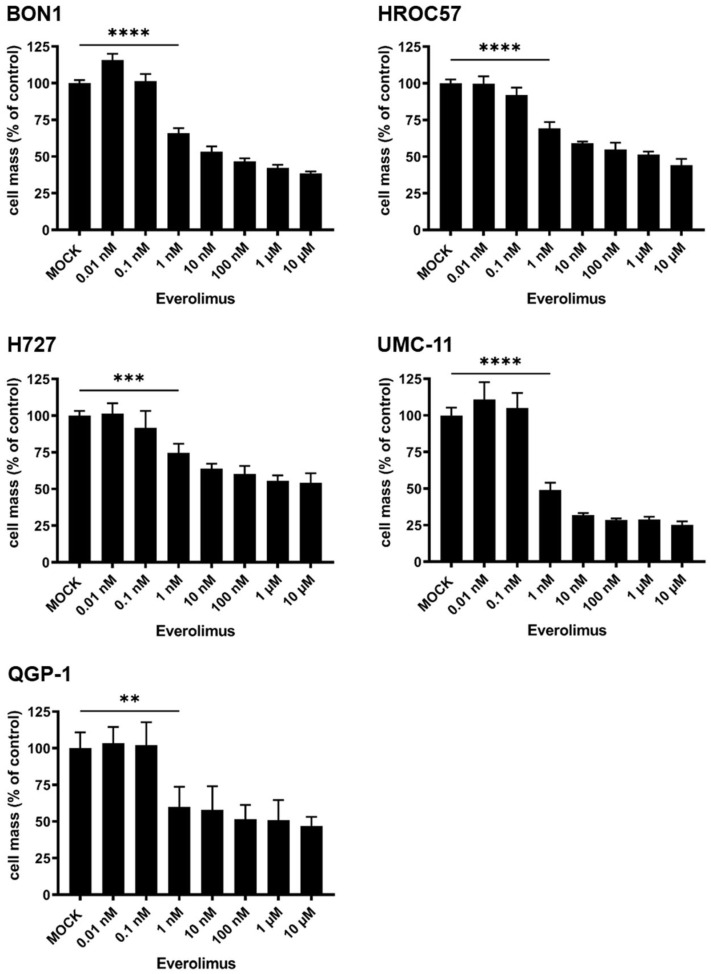
Susceptibility of human NET/NEC cells to mTOR-inhibitor everolimus. The NET/NEC tumor cell lines BON1, HROC57, H727, UMC-11, and QGP-1 were treated with increasing concentrations of everolimus, and tumor cell masses were measured at 96 h post-treatment (hpt) by SRB viability assay. Displayed are mean and standard deviation of two independent experiments performed in triplicate. MOCK: untreated control. ** *p* < 0.01, *** *p* < 0.001, **** *p* < 0.0001.

In this experiment, the different NET/NEC cell lines were treated with rising concentrations of everolimus from 0.01 nM to 10 µM. All tested NET/NEC cell lines responded to everolimus treatment in a dose-dependent manner, with cell masses starting to significantly decrease at a concentration of 1 nM everolimus (Figure 9). Remarkably, BON1 and UMC-11 were the cell lines with the lowest remaining tumor cell mass compared to MOCK-treated controls, more precisely resulting in less than 50% residual tumor mass when using a dose of 10 µM everolimus. Those findings were found to correlate well with results published in the literature [28,48].

#### 3.4.2. Replication Characteristics of MeV-SCD When Combined with Everolimus

**Figure 10:**
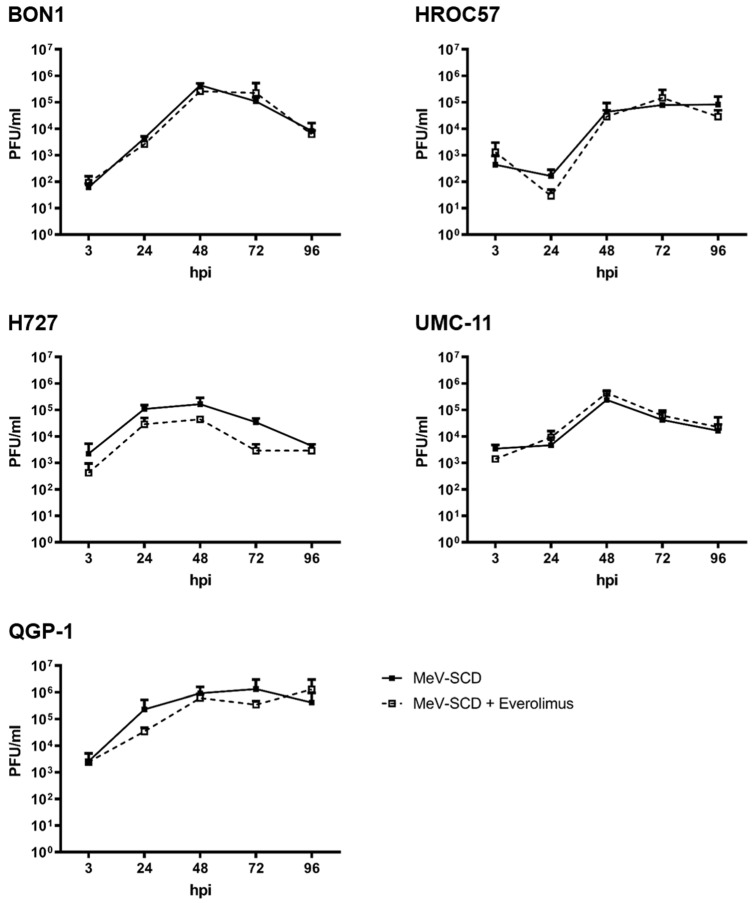
Replication of MeV-SCD in five human NET/NEC cell lines with and without addition of everolimus. The NET/NEC tumor cell lines BON1, HROC57, H727, UMC-11, and QGP-1 were infected with the suicide gene-armed measles vaccine virus MeV-SCD at indicated MOIs, which were adjusted to the respective tumor cell-line-dependent sensitivity. At 3 hpi, the mTOR-inhibitor everolimus was also added in concentrations adjusted to the respective cell line-dependent sensitivity (for all cell lines, 1 nM with the exception of UMC-11, where 0.25 nM was used). Tumor cells and supernatants were harvested at 3, 24, 48, 72, and 96 hpi, and viral titers were determined by TCID_50_ titration. Displayed are mean and standard deviation of two independent experiments performed in duplicate.

For a potential combinatorial therapeutic regime using virotherapy and everolimus for future use in NEN patients, we evaluated the replication kinetics of MeV-SCD in the presence of everolimus in vitro (Figure 10).

MeV-SCD proved to replicate well in all tested NET/NEC cell lines, reaching maximum viral titers between 48 and 72 hpi, while the maximum differed from cell line to cell line. The addition of the mTOR inhibitor everolimus neither impaired nor enhanced viral replication (Figure 10), thus paving the way for future use in patients.

## 4. Discussion

OVs comprise a new treatment option to combat various tumors in advanced stages of disease. Ideally, OVs will selectively (i) infect, (ii) replicate in, and ultimately (iii) oncolyze tumor cells, thereby spearing healthy tissue and minimizing unwanted side effects. Nowadays, there are a variety of OVs used in clinical studies for the treatment of tumors [12]. However, it is important to find the most appropriate OV for the tumor of interest to ensure the best possible treatment for the individual patient, thereby paving the way for future personalized virotherapy.

MeV is one of those viruses that inherit a natural tumor tropism, making it a suitable candidate for various tumor entities [19], including neuroendocrine neoplasms.

In this paper, we used an enhanced, state-of-the-art genetically modified oncolytic MeV encoding the suicide gene SCD, which is a fusion protein of cytosine deaminase and uracil phosphoribosyltransferase (MeV-SCD). It has been shown in our laboratory to be highly efficient in the oncolysis of multiple different solid and “liquid” tumors [32,33,34]. Moreover, resistance phenomena observed to MeV-SCD-mediated oncolysis in tumor cells were successfully overcome by exploiting its suicide gene function by “simple” addition of the prodrug 5-FC, which is then locally converted into the well-known chemotherapeutic agent 5-FU.

Neuroendocrine neoplasms display a very heterogeneous group of different tumors, which urgently require new and effective treatment options due to a rising incidence as well as poor prognosis in advanced stages. While several oncolytic viruses have been tested for their efficiency in neuroendocrine neoplasms [20,21], to the best of our knowledge, MeV vectors have not been evaluated as a potential oncolytic virotherapeutic agent for NENs yet. Therefore, this paper aims to analyze MeV-SCD´s oncolytic potential in a panel of five well-characterized human NEN cell lines derived from different anatomic origins.

The main route of infecting cells for MeV Edmonston vaccine strains is via the so-called CD46 receptor, which is ubiquitously expressed on all nucleated cells. Studies indicate that CD46 receptor density, expressed as the so-called MFI parameter, correlates progressively with viral entry, albeit a distinct threshold is required for cell-to-cell fusions, which ultimately leads to syncytia formation and cell death [46]. Four out of five tested NEN cell lines revealed MFI values >20, which is considered the threshold for cell-to-cell fusion, while only the pancreas-originating cell line QGP-1 showed an MFI underneath the threshold. In this context, we observed primary infection rates of at least 40% up to 80% in all tested cell lines. Notably, the two pancreatic cell lines, QGP-1 and UMC-11, which had the lowest CD46 receptor densities, also exhibited the lowest primary infection rates. Moreover, we proved by immunoblot analysis that the encoded protein SCD is expressed in infected NEN cells, allowing its exploitation in the following experiments. Again, the intensity of the protein band differed remarkably between the different cell lines. In particular, the cell lines with the highest CD46 density and consequently the highest primary infection rates also showed the most intense bands.

After showing that MeV-SCD can successfully infect the five human NEN cell lines, we evaluated its oncolytic efficacy, without exploiting its suicide function. MeV-SCD proved to reduce tumor cell mass in an MOI-dependent manner in all tested cell lines, while the susceptibility varied between the different cell lines. Analogously to other authors [32], we defined cell lines with less than 50% remaining tumor cell mass at 96 hpi when using an MOI of 1 as susceptible, more than 50% as partially resistant, while remaining tumor cell masses of more than 75% were classified as resistant to MeV-mediated oncolysis.

Four out of five NET/NEC cell lines were found to be susceptible to MeV-mediated oncolysis according to our chosen criteria. Not unexpectedly, cell line QGP-1 was found to be partially resistant, probably because of lower infection rates due to a lower CD46 receptor density. However, when using higher MOIs, statistically significant tumor cell mass reduction was observed in all cell lines.

Moreover, we also evaluated the effects of 5-FU monotherapy on the different NEN cell lines, a common chemotherapeutic drug that is still clinically used in the treatment of NENs [10]. All cell lines proven to be highly susceptible to 5-FU in a dose-dependent manner, while the effect was not as pronounced in QGP-1 cells as in other cell lines.

Taken together, both monotherapeutic treatment strategies were shown to reduce tumor cell masses in a dose-dependent manner, thereby encouraging the combination of the two by exploiting MeV-SCD´s inherent suicide gene function.

Furthermore, we evaluated the effect of everolimus monotherapy on our NEN cell line panel. Everolimus is an mTOR inhibitor that is approved for the treatment of a broad spectrum of different NENs, therefore making it an interesting combination partner with OVs. All NEN cell lines were found to be susceptible to everolimus in a dose-dependent manner. These results correlate well with other data published regarding everolimus for the treatment of NEN in vitro [28,48]. While both 5-FU and MeV-SCD were able to toxify all NEN tumor cells when using a sufficiently high dose, there was a threshold for remaining tumor cells when treating them with everolimus: employment of an increased dose did not lead to further tumor cell mass reductions. This is thought to be due to everolimus being an antiproliferative but not a cytotoxic drug by itself.

When studying the viral growth kinetics of MeV-SCD in the presence and absence of everolimus in NEN tumor cells, we did not observe altered virus growth. Interestingly, in another study using the oncolytic vaccinia virus GLV-1h68, everolimus also neither impaired nor enhanced viral replication in NEN cells [29]. The same results were obtained for the later-discussed oncolytic herpes simplex virus talimogene laherparepvec (T-VEC) [28].

Nevertheless, these experiments were conducted solely in monolayer cell cultures. Everolimus, however, is known to have pleiotropic effects on various cell types, including the suppression of the immune system; so, its effect when combining it with OVs needs to be investigated further using in vivo models, especially since there is some evidence that the combination of an mTor-inhibitor, in this case rapamycin, can enhance oncolytic virotherapy both in vitro and in vivo [49,50]. The authors attribute the enhanced viral replication and consecutive enhanced killing of tumor cells to the effects of rapamycin on the innate immune system; in this context, they observed a diminished recruitment of CD68+ natural killer cells [49], a finding analogous to another virus that showed enhanced replication in the presence of rapamycin in vivo [51]. Since we only expect a profound advantage from the combination therapy of MeV-SCD and everolimus in vivo due to its effects on the immune system, we here wanted to exclude a potential general inhibitory effect on replication in vitro. Considering the mechanism of action of MeV-SCD as an immunotherapeutic [19] and the pleiotropic effect of everolimus on the immune system, especially on T cells, an investigation of the combinatorial effect of MeV-SCD and everolimus in tumor/T-cell co-cultures, such as tumor organoid T-cell co-cultures [52], may be helpful and will be considered in future work.

As mentioned above, MeV-SCD has been extensively screened due to its natural tumor tropism, e.g., with the NCI-60 panel. With the same chosen resistance criteria as for this paper, only 50% of the screened cell lines from the panel were shown to be susceptible, while 39% showed a partial and 11% a high-grade resistance, with an average remaining tumor cell mass of 31% at 96 hpi when using an MOI of 1 [32].

In a study of eight sarcoma cell lines, five were found to be permissive, with one cell line showing a partial resistance and two cell lines displaying high-grade resistance to MeV-mediated oncolysis [34]. This highlights the potential of MeV-SCD for the treatment of NEN.

The combination of MeV-SCD and 5-FU was evaluated here by exploiting MeV-SCD’s suicide gene function. The addition of the prodrug 5-FC led to statistically significant further tumor cell mass reductions in all five tested NEN cell lines. Most importantly, the partially resistant NEN cell line QGP-1 now showed a remaining tumor cell mass of less than 50% when using an MOI of 1, thereby displaying that combating NENs with an armed second-generation oncolytic measles virus can overcome partial resistance phenomena.

To further evaluate growth kinetics, we also analyzed the replication characteristics of MeV-SCD in NEN cells in the presence and absence of 5-FC. Interestingly, we observed fewer viral particles when combining MeV-SCD infection with 5-FC, despite achieving enhanced oncolytic activities. Of note, a similar phenomenon was observed with a genetically modified vaccinia virus using the same prodrug-converting system in several cancer cell lines, highlighting that the reduced replication of viral progenies in the presence of 5-FC occurs for both RNA and DNA viruses [53]. The effect is likely caused by both (i) reduced tumor cell masses due to the combined cytotoxic effect of MeV-SCD and converted 5-FU, while tumor cells are mandatory for viral replication, and (ii) direct inhibition of viral replication by incorporation of 5-FUTP into the viral RNA genome. It is likely that both mentioned factors play a role in the reduced MeV-SCD replication in the experiments performed here, but the extent to which each factor plays a role and whether there are other factors as well need to be considered and require evaluation in further experiments.

As mentioned above, different viruses have been tested for the treatment of NENs [20,21]. The three most current investigated viruses are T-VEC [28] and GLV-1h68 [29] in our laboratory, as well as the oncolytic adenovirus “AdVince” [26].

While T-VEC was capable of reducing tumor cell masses at very low MOIs in some cell lines, one NEC cell line was observed that showed partial resistance to T-VEC-mediated oncolysis. Another benefit of T-VEC is that it encodes the transgene GM-CSF as a payload, which could potentially trigger a strong inflammatory antitumoral response in vivo, which could not be observed in this setting. Moreover, T-VEC has a potential safety compound with ganciclovir, which is a virostatic drug inhibiting replication of the virus [28].

AdVince, which constitutes a triple-modified adenovirus currently being tested in a phase I/II clinical trial (NCT02749331) for the treatment of NENs, was investigated in other NEN cell lines and required MOIs of 1 to 10 to achieve sufficient tumor cell mass reduction in vitro [26].

Taken together, T-VEC requires low MOIs for NET cell lines and has the advantage of being a possible antiviral agent if needed. However, due to resistance phenomena observed in NEC cell lines, other viruses such as MeV-SCD, which was exemplarily shown to be able to overcome resistance phenomena by exploiting its suicide gene function, might be the preferential candidate for OV-resistant NEN cells.

From a clinical perspective, ideally, a patient’s tumor should be screened pre-therapeutically for the optimal virotherapeutic drug employing a so-called “virogram”, which evaluates the preferential oncolytic virus in vitro analogously to the well-known antibiogram, helping to find the optimal antibiotic treatment for the respective bacteria [54].

Furthermore, many cancer therapy regimens use a multimodal approach. Likely, oncolytic virotherapy can be further enhanced when combining it with other hallmarks of cancer therapy, such as targeted small molecules (e.g., everolimus) or immune checkpoint inhibitors. Such combination therapies are currently assessed in over 100 different clinical trials [12] and may also represent important aspects of future NEN regimens.

## 5. Conclusions

Here, it was successfully shown for the first time that virotherapy with the measles vaccine virus could be a promising therapeutic option for NEN patients in the future. Especially when using second-generation OVs such as MeV-SCD, which showed a significantly increased oncolytic efficiency in vitro due to its additionally implemented suicide gene function, making it possible to even overcome resistance to OV monotherapy.

## Data Availability

Data are contained within the article and Supplementary Materials.

## Supplementary Materials

The following supporting information can be downloaded at: https://www.mdpi.com/article/10.3390/cancers16030488/s1, Figure S1: Raw data derived from immunoblot analysis of Vinculin in human NET/NEC tumor cell lines; Figure S2: Raw data derived from immunoblot analysis of MeV-encoded SCD protein expression in human NET/NEC tumor cell lines HROC57, UMC-11 and QGP-1; Figure S3: Raw data derived from immunoblot analysis of MeV-encoded SCD protein expression in human NET/NEC tumor cell lines BON1 and H727.
