# Supplementary material for: In Vitro Sensitivity of Neuroendocrine Neoplasms to an Armed Oncolytic Measles Vaccine Virus
Source: Cancers (Basel). 2024 Jan 23;16(3):488. doi: 10.3390/cancers16030488 (PMC10854751; doi:10.3390/cancers16030488)
Supplement: Supplementary file 1 [file cancers-16-00488-s001.zip › Supplementary Figure S1.pdf]

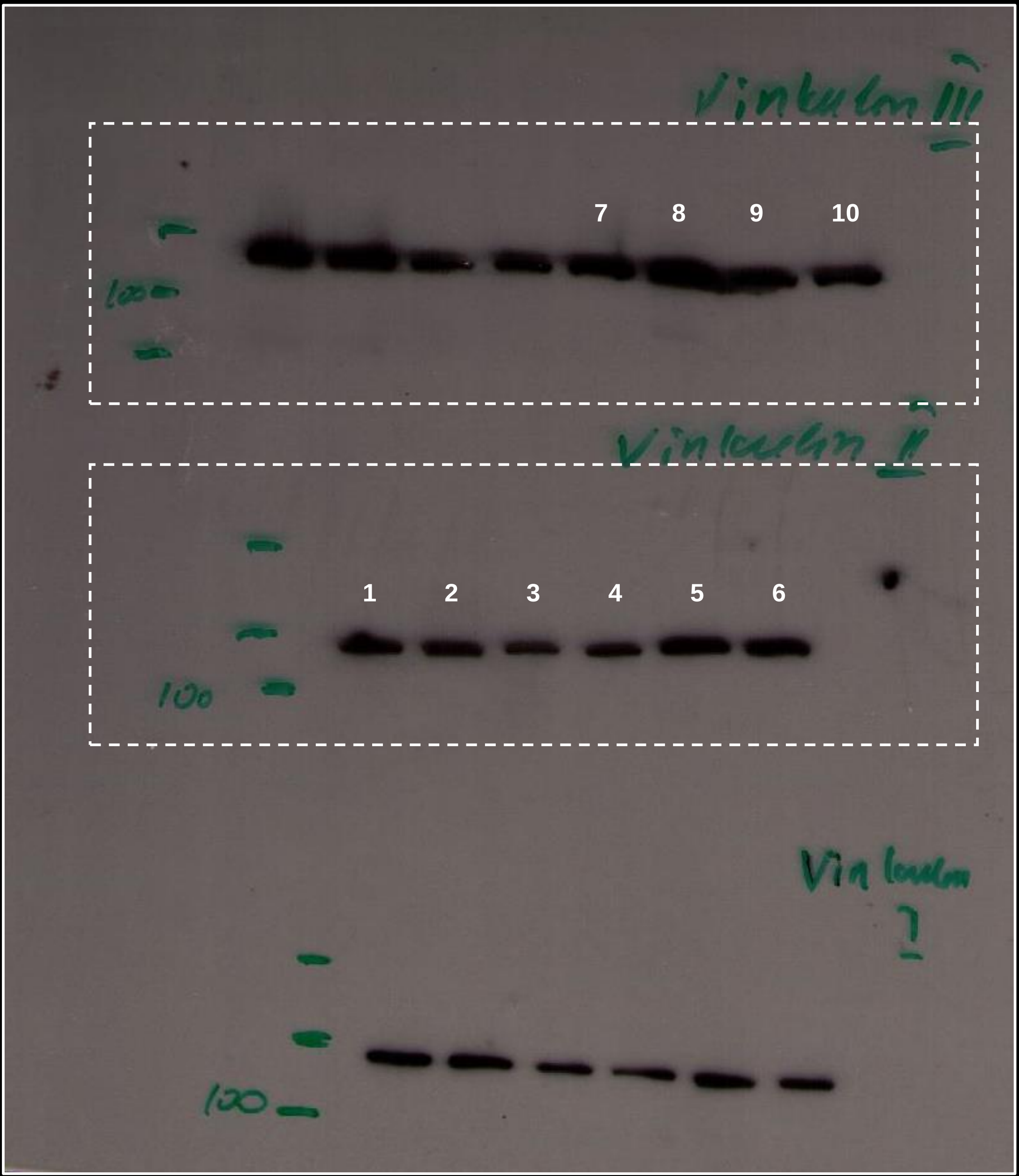

Vinculin bands

1 – HROC57 MOCK

2 – HROC57 MeV-SCD MOI 0.075

3 – UMC-11 MOCK

4 – UMC-11 MeV-SCD MOI 0.5

5 – QGP-1 MOCK

6 – QGP-1 MeV-SCD MOI 1

7 – BON1 MOCK

8 – BON1 MeV-SCD MOI 0.075

9 – H727 MOCK

10 – H727 MeV-SCD MOI 0.75

Figure S1. Raw data derived from immunoblot analysis of Vinculin in human NET/NEC tumor cell lines.
