# Supplementary material for: In Vitro Sensitivity of Neuroendocrine Neoplasms to an Armed Oncolytic Measles Vaccine Virus
Source: Cancers (Basel). 2024 Jan 23;16(3):488. doi: 10.3390/cancers16030488 (PMC10854751; doi:10.3390/cancers16030488)
Supplement: Supplementary file 1 [file cancers-16-00488-s001.zip › Supplementary Figure S2.pdf]

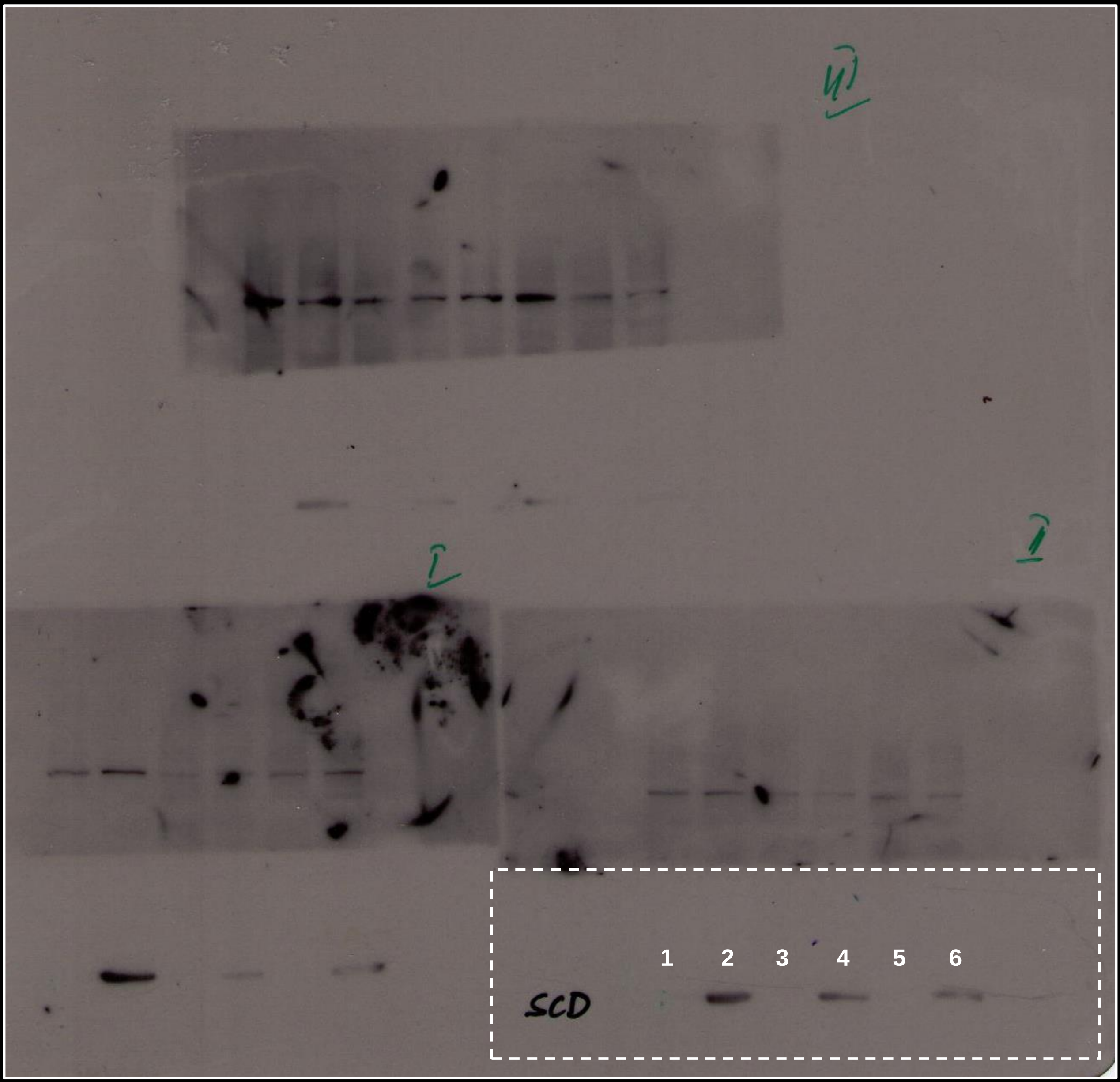

SCD bands

1 – HROC57 MOCK

2 – HROC57 MeV-SCD MOI 0.075

3 – UMC-11 MOCK

4 – UMC-11 MeV-SCD MOI 0.5

5 – QGP-1 MOCK

6 – QGP-1 MeV-SCD MOI 1

Figure S2. Raw data derived from immunoblot analysis of MeV-encoded SCD protein expression in human NET/NEC tumor cell lines HROC57, UMC-11 and QGP-1.
