# Supplementary material for: In Vitro Sensitivity of Neuroendocrine Neoplasms to an Armed Oncolytic Measles Vaccine Virus
Source: Cancers (Basel). 2024 Jan 23;16(3):488. doi: 10.3390/cancers16030488 (PMC10854751; doi:10.3390/cancers16030488)
Supplement: Supplementary file 1 [file cancers-16-00488-s001.zip › Supplementary Figure S3.pdf]

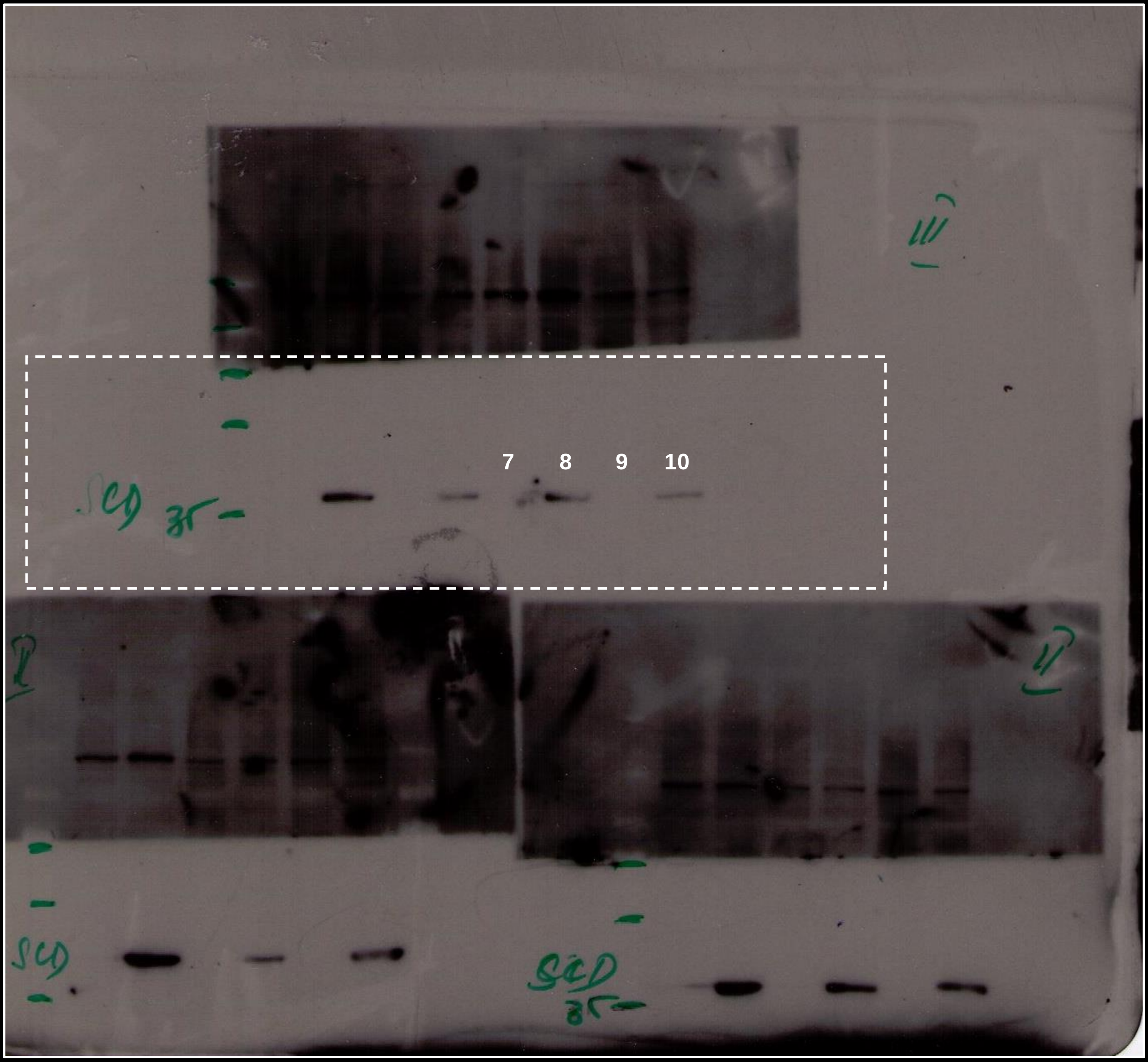


<u>SCD bands</u>

7 – BON1 MOCK

8 – BON1 MeV-SCD MOI 0.075

9 – H727 MOCK

10 – H727 MeV-SCD MOI 0.75

**Figure S3. Raw data derived from immunoblot analysis of MeV-encoded SCD protein expression in human NET/NEC tumor cell lines BON1 and H727.**
